# Audiovisual integration in depth: multisensory binding and gain as a function of distance

**DOI:** 10.1007/s00221-018-5274-7

**Published:** 2018-04-26

**Authors:** Jean-Paul Noel, Kahan Modi, Mark T. Wallace, Nathan Van der Stoep

**Affiliations:** 1 Neuroscience Graduate Program, Vanderbilt Brain Institute, Vanderbilt University Medical Center, Vanderbilt University, Nashville, TN 37235 USA; 2 Vanderbilt Brain Institute, Vanderbilt University Medical Center, Vanderbilt University, Nashville, TN 37235 USA; 3 Department of Hearing and Speech Sciences, Vanderbilt University Medical Center, Nashville, TN 37235 USA; 4 Department of Psychology, Vanderbilt University, Nashville, TN 37235 USA; 5 Department of Psychiatry, Vanderbilt University Medical Center, Nashville, TN 37235 USA; 6 Department of Experimental Psychology, Helmholtz Institute, Utrecht University, Utrecht, The Netherlands

**Keywords:** Multisensory Integration, Audiovisual, Depth, Space, Temporal-binding window, Gain

## Abstract

The integration of information across sensory modalities is dependent on the spatiotemporal characteristics of the stimuli that are paired. Despite large variation in the distance over which events occur in our environment, relatively little is known regarding how stimulus-observer distance affects multisensory integration. Prior work has suggested that exteroceptive stimuli are integrated over larger temporal intervals in near relative to far space, and that larger multisensory facilitations are evident in far relative to near space. Here, we sought to examine the interrelationship between these previously established distance-related features of multisensory processing. Participants performed an audiovisual simultaneity judgment and redundant target task in near and far space, while audiovisual stimuli were presented at a range of temporal delays (i.e., stimulus onset asynchronies). In line with the previous findings, temporal acuity was poorer in near relative to far space. Furthermore, reaction time to asynchronously presented audiovisual targets suggested a temporal window for fast detection—a range of stimuli asynchronies that was also larger in near as compared to far space. However, the range of reaction times over which multisensory response enhancement was observed was limited to a restricted range of relatively small (i.e., 150 ms) asynchronies, and did not differ significantly between near and far space. Furthermore, for synchronous presentations, these distance-related (i.e., near vs. far) modulations in temporal acuity and multisensory gain correlated negatively at an individual subject level. Thus, the findings support the conclusion that multisensory temporal binding and gain are asymmetrically modulated as a function of distance from the observer, and specifies that this relationship is specific for temporally synchronous audiovisual stimulus presentations.

**Electronic supplementary material:**

The online version of this article (10.1007/s00221-018-5274-7) contains supplementary material, which is available to authorized users.

## Introduction

Our senses are equipped with an array of transducers capable of converting different forms of environmental energy into neural signals (e.g., photons impinging on the retina in the case of vision, sound waves moving the cochlear membrane in the case of audition). Information from the different senses is then to be integrated in the central nervous system to build a unified perceptual representation of the world (Calvert et al. 2004; Murray and Wallace 2012). This process of multisensory integration (MSI) has been shown to result in a panoply of behavioral benefits, such as faster and more sensitive perceptual discrimination, as well as more accurate and precise localization of stimuli in space (e.g., Alais and Burr 2004; Diederich and Colonius 2004; Ernst and Banks 2002; Frassinetti et al. 2002; Frens et al. 1995; Lovelace et al. 2003; Noel and Wallace 2016; Nozawa et al. 1994).

The principles governing MSI, originally demonstrated at the level of single neurons, have been found to apply at various levels of neural description (e.g., single units, local field potentials, electroencephalography, functional magnetic resonance imaging, and in behavior and perception, e.g., Meredith and Stein 1986a, b; Wallace et al. 1992; Stein and Meredith 1993; Cappe et al. 2012; though see; Stanford and Stein 2007; Spence 2013). Among these principles, the spatial and temporal principles state that the closer in space and/or time two unisensory stimuli are from one another, the greater the likelihood that these stimuli will be integrated. Although these principles have mainly been studied independently, recent neurophysiological and psychophysical studies have begun to examine the *interactions* between the spatial and temporal aspects of multisensory processing (e.g., Royal et al. 2009, 2010; Slutsky and Recanzone 2001; Stevenson et al. 2012; Zampini et al. 2003). These studies, although representing an important next step toward a comprehensive understanding of the spatiotemporal characteristics governing multisensory processes, remain in their infancy. The importance of this is underscored by the simple observation that manipulating the spatial or temporal features of stimuli in isolation differs from most real-world circumstances, where these features are heavily interrelated and continuously changing.

An important yet often ignored aspect of multisensory processing that strongly relates to the spatial and temporal properties of multisensory processing is the depth or distance of the stimuli (see Van der Stoep et al. 2015a, 2016a for reviews). Indeed, the fact that we perceive and act on stimuli at varying distances poses an interesting challenge for the nervous system. For example, increasing the distance of an audiovisual stimulus pair decreases the intensity of these signals, increases their temporal disparity relative to one another (due to the different propagation speeds of light and sound), and decreases the retinal image size of the visual stimulus. Thus, distance-dependent modulations of multisensory stimuli can dramatically change the spatial and temporal characteristics of these signals, with likely impact on how the brain integrates this sensory information.

Consequently, a question of considerable interest is whether the nervous system can deal with distance-related changes in the spatiotemporal structure of multisensory stimuli when judging the causal structure (e.g., simultaneity) of auditory and visual events. One possible way this could be done is through dynamically changing the timing over which stimuli are bound and integrated (i.e., an alteration in the size of temporal-binding windows, TBW; Stevenson et al. 2016; Noel et al. 2015a, 2016a, b, 2017a, b; De Niear et al. 2017). Alternatively, compensation for the changing spatiotemporal characteristics of the stimuli may be accomplished by shifting the temporal asynchrony that is considered most synchronous (i.e., an alteration in the point of subjective simultaneity, PSS). Finally, both the TBW and PSS may be malleable as a function of stimulus-observer distance. To date, no clear picture has emerged as to how the distance of audiovisual events affects multisensory temporal perception. For example, conflicting findings exist regarding depth-mediated changes in the PSS for auditory and visual signals. Whereas some groups have reported that there may be perceptual compensation for distance evidenced by a shift of the PSS (Alais and Carlile 2005; Engel and Dougherty 1971; Heron et al. 2007; Kopinska and Harris 2004); others have failed to demonstrate such compensation (Arnold et al. 2005; Lewald and Guski 2003).

More recently, a distance-related alteration in multisensory temporal function has been described not via changes in the PSS but through changes in the temporal window within which stimuli are most likely to be reported as synchronous (Noel et al. 2016a). In their study, Noel and colleagues investigated how the TBW changed as a function of whether audiovisual events occurred within or beyond peripersonal space—the space immediately adjacent to and surrounding your body (Serino et al. 2015, 2017). The results suggested that TBWs were larger when stimuli were presented within peripersonal as opposed to extrapersonal space. Similarly, multisensory temporal acuity has been shown to be poorer when a participant’s hand is close rather than far from stimuli (Corveleyn et al. 2015; see also; De Paepe et al. 2014; Parsons et al. 2013).

Other studies have focused on how varying depth modulates the response gain resulting from multisensory integration. More specifically, by measuring simple reaction times to unisensory and multisensory stimuli, multisensory response enhancement (MRE) and race model inequality (RMI) violations^1^ may be measured. Using such approaches, multisensory interactions involving tactile stimulation of the skin have been shown to be largest for stimuli presented in peripersonal as compared to extrapersonal space (e.g., Rizzolatti and Fogassi 1997; Occelli et al. 2011; Noel et al. 2015a, b; Galli et al. 2015; Serino et al. 2015; Salomon et al. 2017). In contrast, multisensory interactions between the exteroceptive sensory modalities (e.g., audition and vision) appear to be largest for stimuli that are presented relatively far (e.g., 200 cm) as opposed to close (e.g., 80 cm) from the observer (Van der Stoep et al. 2016b). These findings, hence, highlight that stimulus-observer distance impacts various facets of multisensory integration, including its temporal constraints and the degree of gain that can be achieved.

Given this prior work, we hypothesized that there may be a relationship between the distance-dependent changes in the temporal range over which one is likely to bind audiovisual information (as indexed via the TBW) and the degree of benefit that one can obtain from binding this information (i.e., multisensory gain). The specific predictions are that broader temporal tuning at near distances would be associated with decreased multisensory gain, and conversely that narrower temporal tuning at far distances would be associated with greater multisensory gain. To test this hypothesis, we had participants perform both a simultaneity judgment (SJ) task and a multisensory redundant target (MRT) task in both near and far space. In each task, we manipulated both the distance from the observer at which stimuli were presented (near vs. far) and the stimulus onset asynchrony (SOA) between the auditory and visual stimuli to investigate multisensory temporal acuity and the temporal profile of multisensory gain.

## Methods

### Participants

Thirty-four (17 females, mean age = 21.34 years, range = 18–26 years) right-handed students from Vanderbilt University took part in this experiment. The sample size was determined via G*Power 3.1 software and based on the effect size from prior studies from our groups (Van der Stoep et al. 2016b; Noel et al. 2016a). The alpha was set at 0.05, and a desired power (1 − *β*) was set at 0.9. The results from two participants were not fully analyzed as their reports of synchrony (see below) did not reach 50% for any SOA. Therefore, the final data set was comprised of 32 participants (16 females, mean age = 21.11 years, range = 18–26 years**)**. All participants reported normal or corrected-to-normal visual acuity, as well as normal hearing. Informed consent was obtained from all individual participants included in the study. The participants were remunerated with class participation credit. The protocols of the study were approved by Vanderbilt University Medical Center’s Institutional Review Board.

### Materials and apparatus

**Fig. 1:**
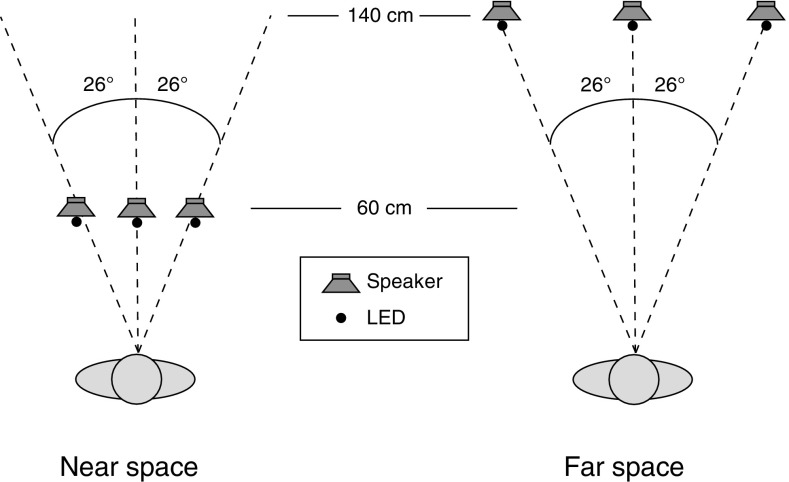
Schematic bird’s-eye-view of the experimental setup. A, V, and AV stimuli were presented in the periphery or centrally in near (left) and far (right) space

Auditory (A), visual (V), and audiovisual (AV) stimuli were presented in near (60 cm) or far (140 cm) space, in a blocked manner (see below). Stimuli could be presented at the periphery (26° to the left or the right of a central fixation) or at fixation (center, 0°; see Fig. 1). Auditory stimuli consisted of a 40 ms pure tone at 3.0 kHz [75 dB(A) at 0.3 m; cosine ramp up and down of 2 ms). Visual stimuli were presented by means of blue Light Emitting Diodes (LED; 5 mm diameter, 465 nm wavelength, 6000 mcd, 34° radiance angle) with a duration of 40 ms. Audiovisual stimuli consisted of the combined presentation of the unisensory A and V stimuli. On AV trials, the unisensory stimuli were presented with a variable SOA of ± 350, ± 250, ± 150, ± 50, or 0 ms (negative SOAs indicate audio-lead, while positive SOAs indicate visual-lead conditions). AV stimuli were always presented spatially aligned. The A and V stimuli were not corrected for intensity or retinal image size across distance as it was recently demonstrated that audiovisual integration is enhanced for stimuli that are presented in far as compared to near space when stimuli are not corrected, and here, we attempt to replicate and extend these findings (see Van der Stoep et al. 2016b for the effects of stimulus effectiveness and changes in distance on audiovisual integration).

### Procedure

Participants were comfortably seated in a dimly lit room. During separate blocks, participants performed either the multisensory redundant target (MRT) or the simultaneity judgment (SJ) task with stimuli presented in either near or far space. Block order, both in terms of the task performed (MRT and SJ task) and distance (near or far), was counter-balanced across participants.^2^

#### Simultaneity judgment task

In the SJ task, only AV stimuli were presented. Participants were instructed to fixate at the central LED and to report whether an audiovisual event was synchronous or asynchronous using two buttons. A total of 675 trials were presented per distance: 60 trials per SOA at the peripheral locations (30 left, 30 right), and 15 trials per SOA at the central location. Inter-trial interval was random between 1200 and 2200 ms (uniform distribution).

#### Multisensory redundant target task

In the case of the MRT task, A, V, and AV targets were presented in random order. At the start of each trial, participants were to fixate at the central LED. Then, they performed a Go/No-go detection task, in which they had to respond as fast as possible to peripheral but not central locations by button press (i.e., an implicit spatial discrimination task using multisensory stimuli, see Van der Stoep et al. 2015b, c, 2016b, 2017; see McDonald and Ward 1999 for a description of the original paradigm; see Fig. 1). The chosen paradigm, thus, not only keeps the MRT task in the current study similar to that of Van der Stoep et al. (2016b), but it also makes spatial information task-relevant, allowing for potential differences in spatial uncertainty between near and far space to modulate MSI. At each distance, 550 trials were performed. There were 80 unisensory Go trials (20 left and 20 right targets for both A and V stimuli) and 20 unisensory No-go trials (center, 10 A, 10 V). There were 40 AV Go (20 left, 20 right) and 10 AV No-go (center) trials for each of the nine SOAs (see “Materials and apparatus”). AV stimuli were always presented spatially aligned but could vary in their temporal alignment as described above. The inter-trial interval was random between 1200 and 2200 ms (uniform distribution).

### Analyses

#### Simultaneity judgment task

The proportion of reports of synchrony was calculated for each SOA and each participant. Reports of synchrony for left and right targets were amalgamated, as there was no a-priori reason to postulate a difference between these conditions. These resulting distributions were subsequently fit via the method of least squares with a Gaussian distribution whose amplitude, mean, and standard deviation were free to vary (see Eq. 1; Noel et al. 2015a, 2016a, b, 2017a; Simon et al. 2017):

1 $$P\left( {{\text{response|SOA}}} \right)=~{\text{amp}}~ \times {\text{~ex}}{{\text{p}}^{ - \,\left( {\frac{{{{({\text{SOA}} - {\text{PSS}})}^2}}}{{2{\text{S}}{{\text{D}}^2}}}} \right)}}.$$

The shape of the normal distribution proved to accurately describe the shape of the distribution of the reports of synchrony (near space: group mean *R*^2^ = 0.94, SD = 0.05; far space: group mean *R*^2^ = 0.91, SD = 0.05). For each participant, the mean of the best fitting distribution was taken as the PSS, and the distribution’s standard deviation as a measure of the TBW (see Noel et al. 2015a, 2016a, b; De Niear et al. 2017). The PSS is the SOA at which participants are most likely to categorize an AV presentation as simultaneous. The TBW is the temporal interval over which participants are highly likely to categorize the presentation as synchronous.

#### Multisensory redundant target task

Reaction times for correct trials (responses to Go trials) were first trimmed for responses that were faster than 120 ms and slower than 1000 ms as they were considered to be the result of anticipation or not paying attention to the task, respectively. As a result, a total of 1.7% of the trials for the near condition and 2.1% of the trials for the far condition were removed from further analysis. In the case of multisensory stimuli presented with a non-zero SOA, reaction times (RTs) were computed as the time elapsed between the presentation of the first sensory stimulus and the button press, thus allowing alignment between unisensory and multisensory RTs. RTs for left and right target trials were collapsed. Subsequently, the median response times of each participant in each condition were used in the RT analysis as RT distributions are generally skewed and the median is less affected by the presence of outliers (Whelan 2008).

**Fig. 2:**
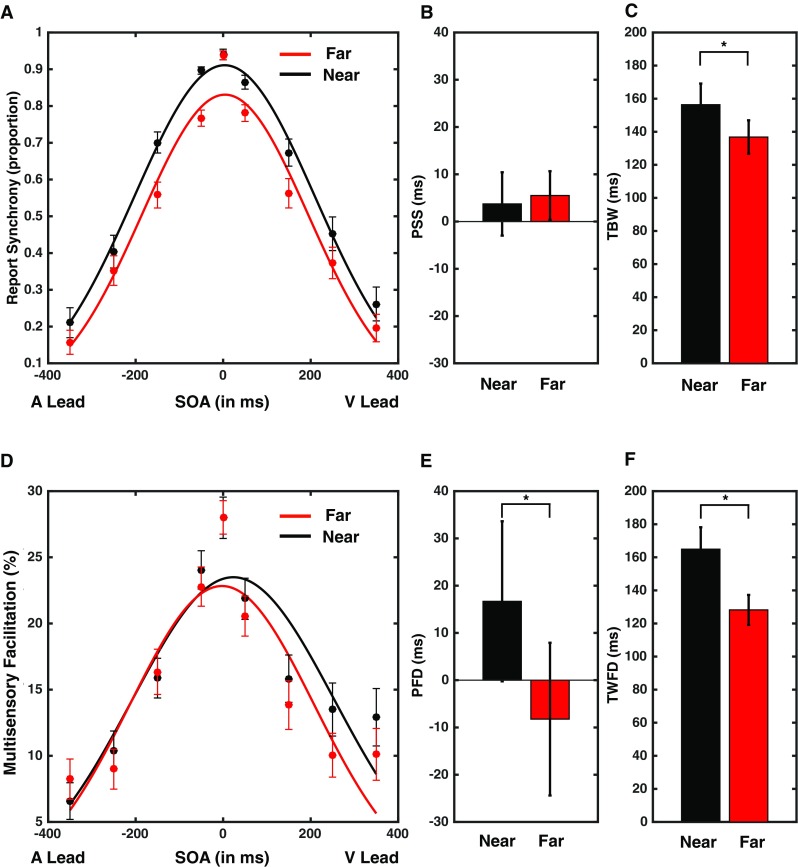
Performance on the SJ and MRT task as a function of SOA for near and far space. **a** Proportion of ‘simultaneous’ responses (i.e., reported synchrony) as a function of SOA and distance at which audiovisual stimuli were presented (black = near; red = far). **b, c** Group average for the PSS and TBW in near (black) and far (red) space. **d** Normalized multisensory gain as a function of SOA for near (black) and far (red) space. **e, f** Group average for the PFD and TWFD in near (black) and far (red) space. Significant differences are indicated with an asterisk (*p* < 0.05). Error bars represent standard error of the mean

To draw an analogy with the TBW obtained from the SJ task analysis (see above), and to better quantify the impact of SOA on multisensory gain, we normalized for each participant and for each distance the median AV RTs at all SOAs by dividing them by the slowest median AV RT (e.g., for one participant, the slowest median AV RT could be the one in the − 350 ms SOA condition; and for another participant in the − 250 ms SOA condition). For all participants except one in the Far condition and two in the Near condition, the slowest multisensory RT was at an asynchrony of either ± 250 or 350 ms (i.e., the largest asynchronies). This normalization procedure resulted in normalized RTs ranging between 0 (fastest responses) and 1 (slowest responses), providing a U-shaped distribution across SOAs. To match the shape of this distribution to that of the SJ task (i.e., an inverted U-shape/Gaussian distribution), we simply subtracted the normalized values from 1 (1-normalized RT). Finally, we multiplied the resultant values by 100 to illustrate the temporal profile of multisensory facilitation in percentage (see Fig. 2d). Thus, using this approach, we obtained a distribution of normalized median AV RTs between 0 (slowest responses) and 100% (fastest responses), indexing multisensory facilitation. Next, a Gaussian distribution was fit to the pattern of normalized RTs (as for the SJ task). The fits proved to be accurate in describing the pattern of multisensory RTs for both the near and far condition (group average near *R*^2^ = 0.72, SD = 0.14, *R*^2^ range = 0.39–0.92, 1 participant rejected due to non-converging fits; group average far *R*^2^ = 0.73, SD = 0.15, *R*^2^ range = 0.26–0.90). To, respectively, mimic the PSS and TBW obtained from the SJ task, the Point of Fastest Detection (PFD) and the Temporal Window of Fast Detection (TWFD) were calculated for each participant and each distance reflecting (1) the estimated SOA resulting in the fastest multisensory RT (mean of the distribution), and (2) the SOA window within which responses were fastest (the standard deviation of the Gaussian fit). Correlations between the PSS and PFD, as well as between the TBW and TWFD were calculated (see Mégevand et al. 2013 for a similar approach).

Subsequently, to investigate multisensory gain, we adopted a two-step approach. Multisensory gain is best indexed by determining whether multisensory RTs are faster than predicted by the race model, as this model indicates whether the gain can be explained by independent processing of the unisensory signals (Miller 1982, 1986, 2015). However, as this analysis involves comparisons at a number of deciles or comparing the shape of distributions of RTs, it is possible to obtain punctuate and spurious race model violations that result from the number of statistical comparisons or from other processes than MSI, such as facilitation due to shifts of crossmodal spatial attention (Van der Stoep et al. 2016a, b). Thus, here, we first index whether multisensory reaction times results in multisensory enhancement according to Eq. (2):

2 $${\text{Relative~MRE}}=\frac{{\hbox{min} \left( {{\text{R}}{{\text{T}}_{{\text{A,\,SOA\,corrected}}}},~{\text{R}}{{\text{T}}_{{\text{V,\,SOA\,corrected}}}}} \right) - {\text{R}}{{\text{T}}_{{\text{AV}}}}}}{{\hbox{min} \left( {{\text{R}}{{\text{T}}_{{\text{A,\,SOA\,corrected}}}},~{\text{R}}{{\text{T}}_{{\text{V,\,SOA\,corrected}}}}} \right)}} \times ~100\% ,$$

where ${\text{R}}{{\text{T}}_{{\text{AV}}}}$ indicates the median RT in multisensory conditions. Importantly, $\hbox{min} \left( {{\text{R}}{{\text{T}}_{{\text{A,\,SOA\,corrected}}}}{\text{,~R}}{{\text{T}}_{{\text{V,\,SOA\,corrected}}}}} \right)$ indicates the fastest unisensory median RT after correction for SOA. That is, in the example of comparing unisensory RTs to a multisensory RT where the auditory stimuli were presented 150 ms prior to visual stimuli, we delayed the unisensory RTv by 150 ms to mimic the multisensory presentation. Overall, therefore, in this first step, we did not explicitly take into consideration the shape of the multisensory and unisensory RT distributions, but simply asked if the RTs at a given distance and SOA exhibited multisensory enhancement when contrasting medians. Next, we queried whether the observed MRE could be explained by statistical facilitation or not, solely at those SOAs demonstrating multisensory enhancement. For those SOAs at which significant rMRE was observed, the cumulative distribution function (CDF) of RTs to A, V, and AV targets was calculated for each distance (near and far). As for the MRE analysis, the unisensory CDFs were first corrected for SOA (see Leone and McCourt 2015; Harrar et al. 2016) and then summed to calculate the upper bound of statistical facilitation predicted by a race model (see Eq. 3; Colonius and Diederich 2017; Miller 1982, 1986, 2015; Raab 1962; Ulrich et al. 2007). Violations of the Race Model Inequality (RMI) indicate an interaction between the senses:

3 $$P\left( {{\text{R}}{{\text{T}}_{{\text{AV}}}}<t} \right) \leqslant P\left( {{\text{R}}{{\text{T}}_{\text{A}}}<t} \right)+~P\left( {{\text{R}}{{\text{T}}_{\text{V}}}<t} \right).$$

The RMI describes the probability of a given RT in the multisensory condition that is less than or equal to a given time *t* based on the combined probabilities for a given RT in the unisensory conditions where *t* ranges from 120 to 1000 ms (assuming a maximum negative correlation of − 1 between processing times of unisensory stimuli). For each participant and each condition, the performance in the AV condition was compared to the upper bound predicted by the race model by comparing RTs (*x*-axis) for a range of deciles (*y*-axis, nine points: 10, 20, up to 90%) of the AV and the race model CDF (i.e., the sum of the A and V CDF). Given the aim of establishing a relationship between multisensory binding and gain as a function of distance, differences in RT between the audiovisual CDF and the race model CDF for each decile (i.e., race model inequality violation) were analyzed using one-tailed pairwise comparisons for each decile and each condition (*p* values were corrected for nine tests in each condition using the Bonferroni method). A central comparison here was whether RMI violations occurred over greater range for far relative to near space. Given the four SOAs demonstrating rMRE (i.e., − 150, − 50, 0, and 50 ms), we performed a 2 (Distance; Near vs. Far) × 4 (SOA; − 150, − 50, 0, 50 ms) × 9 (Deciles) repeated-measures ANOVA to investigate changes in race model violation as a function of distance and SOA.

For completeness, we also present Race Model Inequality (RMI) violations calculated as the probability differences between the AV CDF and the race model CDF (*y*-axis) for each RT (*x*-axis) and for all SOAs in the supplementary materials (including those asynchronies under which MRE was not observed, and in which the violations are likely the result of attentional/task-demand factors, see Figs. S1 and S2. Furthermore, all data are publically available at the Open Science Framework, Noel and Van der Stoep 2017).

## Results

### Simultaneity judgment task

As illustrated in Fig. 2 **(**upper panel**)**, TBWs were significantly smaller (i.e., audiovisual temporal acuity was significantly better) when AV stimuli were presented in far (*M* = 136.34 ms, SE = 10.10 ms) as opposed to near space [*M* = 156.34 ms, SE = 12.77; *t*(31) = 3.01, *p* = 0.004, Fig. 2c]. In contrast, there was no significant difference in the PSS for stimuli presented in far vs. near space [*t*(31) = 0.111, *p* = 0.913; *M*_Far_ = 5.49 ms, SE = 5.14 ms; *M*_Near_ = 3.72 ms, SE = 6.69 ms, Fig. 2b].

### Multisensory redundant target task

#### Accuracy

Overall, participants had a high percentage of hits on Go trials (*M*_A_ = 95%, SE = 1.76%, *M*_V_ = 97%, SE = 1.76%; *M*_AV_ = 99%, SE = 0.35%), while also exhibiting a sizable number of false alarms (responses on No-go trials/central targets, *M*_A_ = 26%, SE = 5.48%; *M*_V_ = 16%, SE = 4.41%; *M*_AV_ = 20%, SE = 5.12%). This high number of false alarms likely resulted from the strong emphasis on speed rather than accuracy, and the fact that Go trials outnumbered No-go trials by a factor of four. Furthermore, participants had a higher proportion of hits on AV when compared with unisensory (e.g., A alone, V alone) trials as indicated by a 3 (Target Modality: A, V, AV) × 2 (Distance: near, far) repeated-measures ANOVA. There was a main effect of Target Modality [*F*(2,58) = 10.72, *p* < 0.001, partial *η*^2^ = 0.27]. Importantly, no main effect of Distance [*F*(1, 30) = 1.63, *p* = 0.22] nor an interaction between Distance and Target Modality [*F*(2,58) = 0.57, *p* = 0.56] was observed.

In terms of false alarms, a 3 (Target Modality: A, V, AV) × 2 (Distance: near, far) repeated-measures ANOVA revealed a main effect of Target Modality [*F*(2,58) = 5.04, *p* = 0.009, partial *η*^2^ = 0.14], yet no main effect of Distance [*F*(1,30) = 0.55, *p* = 0.46] nor an interaction between Target Modality and Distance [*F*(2,58) = 1.81, *p* = 0.10]. Post hoc analyses (paired t test) indicated more false alarms on A trials than on V trials [*t*(31) = 2.99, *p* = 0.005, Cohen’s *d* = 0.59] or AV [*t*(31) = 2.23, *p* = 0.033, Cohen’s *d* = 0.34]. There was no significant difference in false alarms on V and AV trials [*t*(31) = 1.95, *p* = 0.059, Cohen’s *d* = 0.27].

These results reflect the emphasis on ‘speed over accuracy’ in the instructions and importantly indicate that there was no difference in response accuracy for the main factor of interest: Distance. Thus, the remainder of the analyses for the RTE task were focused on the pattern of response times (RTs).

### Response times

#### The temporal profile of normalized multisensory gain

To investigate the effect of distance on the temporal profile of multisensory gain **(**yet not necessarily the amount, see below), the pattern of multisensory RTs as a function of SOA was analyzed for each distance. Two metrics were calculated in this analysis—the Point of Fastest Detection (PFD) and the Temporal Window of Fast Detection (TWFD)—measures analogous to the Point of Subjective Simultaneity (PSS) and the Temporal-Binding Window (TBW) derived from the SJ task (see “Methods” for additional detail). The TWFD was significantly smaller when stimuli were presented in far (*M* = 138.21 ms, SE*M* = 9.0 ms) as compared to near space (*M* = 164.95 ms, SD = 13.22 ms, *t*(31) = 2.05, *p* = 0.04, see Fig. 2d, f). In addition, the PFD was significantly different between near and far space [*t*(31) = 3.51, *p* = 0.001; near: *M* = 16.8 ms, SE = 16.93 ms; Far: *M* = − 8.23 ms, SE = 16.16 ms, Fig. 2e], indicating that maximum multisensory gain (as indexed via speeding of responses) was obtained for AV stimuli with a small visual-lead in near space, and for those with a small auditory lead in far space. These asymmetries nicely mimic the fact that audition is slower than vision in medium (and hence at far distances audio-leads would promote temporal co-occurrence), but the auditory neural system is quicker than the visual one (and hence, at near distances, visual-leads would promote temporal co-occurrence).

#### Correlations of the temporal windows across tasks

There was no correlation between the temporal windows derived from the SJ and MRT tasks (TBW and TWFD; MRT near vs. SJ near, *r* = 0.10, *p* = 0.58; MRT far vs. SJ far, *r* = − 0.03, *p* = 0.89) or in the degree to which participant’s performance was affected by distance in the two tasks ([RT close–RT far] vs. [SJ close–SJ far], *r* = − 0.09, *p* = 0.60). Hence, at the group level, analyses of synchrony judgments and RTs as a function of SOA and distance are consistent with the presence of larger temporal windows of multisensory interactions in near as opposed to far space. However, these processes appear to be somewhat independent as there is no correlation between the simultaneity judgment and reaction time task at an individual subject level.

#### Relative multisensory response enhancement

**Fig. 3:**
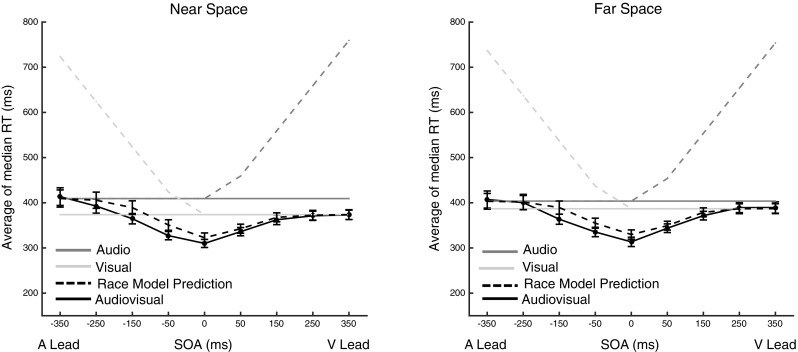
Average of median RTs in the AV condition (solid black line) in near (left panel) and far (right panel) space as a function of SOA between the auditory and visual stimuli. The average of median RTs in response to auditory (dark gray) and visual (light gray) targets are shown for comparison. Similarly, unisensory reaction times corrected for SOA (dashed shaded lines) are plotted for completeness. Finally, averaged median race model predictions are plotted (black dashed line) given the unisensory median reaction times corrected for SOA. Error bars indicate standard error of the mean

Next, we analyzed relative MRE (rMRE; the percentage multisensory gain relative to the fastest unisensory RT) as a function of distance and SOA. The two (Distance: near vs. far) × 9 (SOAs) repeated-measures ANOVA demonstrated a main effect of SOA [*F*(8,248) = 38.15, *p* < 0.001] but no SOA × Distance interaction [*F*(8,248) = 0.73, *p* = 0.66], nor a main effect of Distance [*F*(1,31) = 0.013, *p* = 0.900]. Separate one-sample *t* tests (comparison with zero/no facilitation) showed significant rMRE in both near and far space for the − 150 ms (near, *M* = 7.73%, SE = 1.57, *t*(31) = 3.77, *p* < 0.001; far, *M* = 8.04%, SE = 1.58, *t*(31) = 5.08, *p* < 0.001), the − 50 ms (near, *M* = 13.13%, SE = 1.53, *t*(31) = 8.54, *p* < 0.001; far, *M* = 12.35%, SE = 1.17, *t*(31) = 10.47, *p* < 0.001), the 0 ms (near, *M* = 12.75%, SE = 1.29, *t*(31) = 9.86, *p* < 0.001; far, *M* = 14.09%, SE = 1.45, *t*(31) = 9.70, *p* < 0.001), the + 50 ms (near, *M* = 9.07%, SE = 0.81, *t*(31) = 11.12, *p* < 0.001; far, *M* = 9.71%, SE = 1.20, *t*(31) = 8.06, *p* < 0.001), and the + 150 ms (near, *M* = 2.89%, SE = 1.03, *t*(31) = 2.78, *p* < 0.001; far, *M* = 3.57%, SE = 1.21, *t*(31) = 2.95, *p* < 0.001) conditions. There were no significant differences between near and far space at any SOA (all *p* > 0.23). In sum, multisensory enhancement is seemingly present solely at relatively small asynchronies (= < 150 ms). A similar pattern of results emerges when scrutinizing absolute, as opposed to relative, multisensory enhancement (i.e., Eq. 2 without the denominator; see supplementary material online) (Fig. 3).

#### Race model inequality violation

Finally, using race model inequality analyses, we investigated whether rMRE could be explained in terms of statistical facilitation (i.e., independent channels) or involved an active integration of information across the different sensory channels. Even though the amount of rMRE was similar across distances, this analysis was used to determine whether the amount of RMI violation was different between near and far space.

**Fig. 4:**
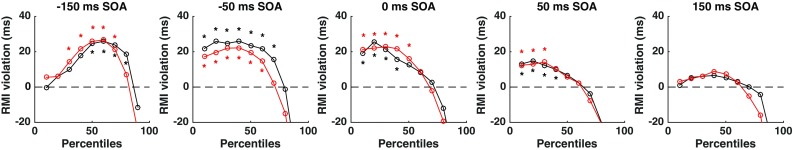
Race model inequality violations (in ms) for responses to near (black) and far (red) audiovisual stimuli at SOAs of − 150 (leftmost) − 50 (second), 0 (third), and 50 (rightmost) ms. Significant violations are indicated with an asterisk (*p* < 0.05 corrected for nine tests using the Bonferroni method)

As illustrated in Fig. 4, significant race model violations were observed for SOAs ranging from − 150 to 50 ms in both near and far space. For the true synchrony condition (0 ms SOA), significant violations were observed from the 10th to the 40th percentile in near space and from the 10th to the 50th percentile in far space (all *p* < 0.05). However, statistically contrasting the amount of RMI violations between the near and far space at SOA = 0 ms via a paired *t* tests suggested no significant difference in the amount of race model violation between distances (all *p* > 0.26, 4th decile). In the case of SOA = − 50 ms, the race model was violated across a range spanning the first to seventh decile when stimuli were presented in the near space, and between the range spanning the first and sixth decile when audiovisual stimuli were presented in the far space. In the case of SOA = 50 ms, the race model was violated between the first and fourth decile in the near space, and between the first and third decile in the far space. Finally, in the case of SOA = − 150 ms, the race model was violated between the third and seventh decile in the near space and between the fifth and eighth decile in the far space. Nonetheless, as in the case of synchronous presentation, the contrast between RMI violations in near and far space was not statistically significant for SOA = − 150 ms (all *p* > 0.18, 8th decile), SOA = − 50 ms (all *p* > 0.10, 7th decile) nor SOA = 50 ms (all *p* > 0.28, 9th decile). Finally, a two (Distance; Near vs. Far) × 4 (SOA; − 150, − 50, 0, 50 ms) × 9 (Decile) repeated-measures ANOVA on RMI violations demonstrated a significant main effect of SOA [*F*(2,93) = 12.99, *p* < 0.001, partial *η*^2^ = 0.29] and Decile [*F*(8,248) = 54.06, *p* < 0.001, partial *η*^2^ = 0.63], as well as a significant SOA × decile interaction [*F*(24,744) = 16.06, *p* < 0.001, partial *η*^2^ = 0.34]. Importantly, there was no main effect, nor any interaction with Distance (all *p* > 0.46).

#### Correlation between changes in temporal-binding window and the range of RMI violations

**Fig. 5:**
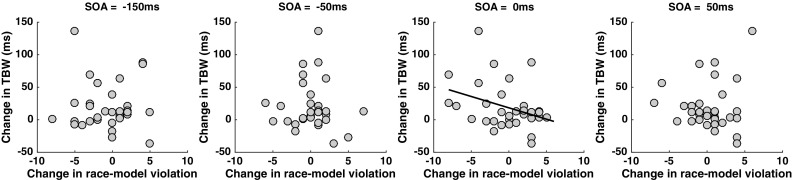
Correlations between the change in TBW between near and far space and the number of deciles violating (> 0) the race model as a function of distance (Near–Far) for SOAs exhibiting a significant race model violation at the group level (leftmost: SOA = − 150 ms; second: SOA = − 50 ms; third: SOA = 0 ms; rightmost: 50 ms). None of these correlations were significant when considered in isolation (SOA = − 150 ms: *r* = − 0.01, *p* = 0.94; SOA = − 50 ms: *r* = − 0.06, *p* = 0.73; SOA = 0 ms: r = − 0.34, *p* = 0.056; and SOA = 50 ms: r = 0.04, *p* = 0.79), but the correlation at SOA = 0 ms is significant (rho = − 0.4, *p* = 0.02) when the co-variance at other SOAs is included in a partial correlation

To test the hypothesis of whether differences in the size of the TBW (as assessed via the SJ task) in near and far space are associated with changes in multisensory gain (as assessed via the MRT task), a correlational analysis was performed. We first correlated the difference in the size of the TBW between near and far space with the difference in the number of deciles (1–9) at which RMI violations were observed for synchronously presented stimuli (0 ms SOA), as well as for stimuli presented with small asynchrony (i.e., ± 50 and − 150 ms). There was no significant correlation between the impact of distance on the TBW size and the number of deciles violating the race model at SOA = − 150 ms (*r* = − 0.01, *p* = 0.94), SOA = − 50 ms (*r* = − 0.06, *p* = 0.73) or SOA = 50 ms (*r* = 0.04, *p* = 0.79), yet a strong trend existed at SOA = 0 ms (*r* = − 0.34, *p* = 0.056, see Fig. 5). Furthermore, when controlling for partial correlations at other asynchronies, the results indicated a significant correlation between the aforementioned variables at SOA = 0 ms (rho = − 0.47, *p* = 0.008, while controlling for variance at SOAs = − 150, − 50 and 50 ms), but not at SOA = − 150 ms (rho = 0.27, *p* = 0.14, while controlling for SOAs = 0, − 50, and 50 ms), SOA = − 50 ms (rho = 0.05, *p* = 0.76, while controlling for SOAs = 0, − 150, and 50 ms) nor SOA = 50 ms (rho = 0.21, *p* = 0.27, while controlling for SOAs = − 150, − 50 and 0 ms).

In sum, while at the group level, TBWs appear to enlarge as stimuli are observed from a closer distance and multisensory gain is relatively stable given the current distances, at an individual subject level there appears to be an asymmetric relationship between multisensory binding and gain. Furthermore, this relation is seemingly specific to the case of multisensory gain during synchronous and not asynchronous presentations.

## Discussion

The aim of the current study was to investigate how changes in the distance and timing from which audio–visual stimuli are presented affect multisensory temporal acuity and gain. Recently, in two separate studies, it was observed that temporal-binding windows are larger in near space (Noel et al. 2016a), while multisensory gain is greater in far space (Van der Stoep et al. 2016b). Therefore, we hypothesized that distance-related decreases in temporal-binding windows would be associated with increases in multisensory gain at the level of the individual subject. To examine this relationship, we had the same participants perform both an audiovisual simultaneity judgment task and an audiovisual redundant target task using the same stimuli presented in both near and far space and at nine different stimulus onset asynchronies. Thus, the results of the current study build upon prior studies by indexing multisensory gain in space at different temporal asynchronies, by indexing temporal-binding windows at distinct stimulus-observer distances, and by indexing multisensory temporal binding and gain within the same participants.

In line with the previous observations, we observed poorer audiovisual temporal acuity (i.e., larger TBWs) for stimuli presented in near relative to far space. It must be highlighted that in addition to supporting prior observations, the current work extends upon them by physically manipulating the position of audiovisual targets in depth—that is, in the previous studies (e.g., Noel et al. 2016a), TBWs were measured either within or beyond the boundary of peripersonal space without physically displacing the stimuli in depth. Furthermore, the results of the multisensory redundant target task enabled the establishment of the novel construct of the temporal window of fastest detection (a reaction time-based analogy to the TBW), and showed that this window is larger in near relative to far space. This latter observation indicates that distance-dependent changes in audiovisual temporal processes are not only applicable to perceptual–decisional phenomena (such as what is done during a simultaneity judgment), but also apply to simple motor responses (such as those measured in a speeded response task). This is an important result as temporal order and simultaneity judgment tasks have been argued to be prone to response biases (Schneider and Bavelier 2003; Van Eijk et al. 2008). In addition, to the best of our knowledge, the current data are the first to demonstrate an increased multisensory temporal tolerance when stimuli are presented in far vs. near space without correcting for retinal image size and stimulus intensity. This way of measuring distance-related changes in multisensory perception makes good ecological sense as changes in stimulus effectiveness and distance generally go hand in hand (i.e., manipulating the stimulus-observer distance alters stimulus properties such as the intensity and retinal image size of stimuli).

Interestingly, while at a group level, both the TBW and TWFD were altered by distance in the same direction (i.e., larger windows in the near space), this was not true for the PSS and PFD. Indeed, while the PSS was unaltered by distance (but see Sugita and Suzuki 2003; Alais and Carlile 2005), the PFD was positive (i.e., visual-lead) when stimuli were presented in near space and negative (i.e., audio-lead) when stimuli were presented in far space. This pattern of results makes good ecological sense, as sound energy travels more slowly than light, and thus, the arrival of auditory stimuli at the cochlea is increasingly delayed relative to the arrival of light energy at the retina with increasing distance. A speculation here is that the simultaneity judgment task involves more cognitive processes than the redundant response task, and hence, the latter may more faithfully mimic statistical regularities of the environment, while during the former participants may employ a number of strategies leading to unaltered simultaneity judgments as a function of distance.

It has previously been shown that multisensory gain is larger for synchronously presented auditory and visual stimuli in far relative to near space (Van der Stoep et al. 2016b). However, in the current study, when contrasting near and far conditions, we did not observe a difference in race model inequality violations at the group level. A putative explanation for the lack of effect of distance on multisensory gain here is the use of a smaller range of distances (60 and 140 cm here vs. 80 and 200 cm in Van der Stoep et al. 2016b) and the use of physically smaller stimuli, resulting in smaller changes in stimulus effectiveness (e.g., intensity and retinal image size) with changing distance in the current study. Given that we also manipulated the SOA at which stimuli were presented, we were also able to detail the temporal profile of multisensory gain. Interestingly, at relatively small asynchronous SOAs (e.g., 150 ms), race model violations were generally observed over a greater range of the response time distribution in near as opposed to far space, although once again, the direct contrasts between near and far space did not reach significance. These results suggest that multisensory gain may be present over a larger range of temporal asynchronies in near than in far space, and that this gain is larger in far relative to near space but only within a very restricted temporal window. While this evidence for an opposing relationship between multisensory binding and gain as a function of distance from the observer is weak at the group level, it is more readily apparent at an individual subject-level. In fact, when accounting for the co-variance between multisensory binding and gain at asynchronous audiovisual presentations, there was a strong negative correlation between the impact of distance on the degree to which participants bound multisensory stimuli in time, and their degree of multisensory gain when stimuli were presented synchronously. A similar relation did not exist when indexing multisensory gain during asynchronous presentations.

Overall, these results are reminiscent of findings by Leone and McCourt (2013, 2015), who manipulated auditory and visual stimulus intensity, as well as stimulus timing, to measure the effects of physical and physiological simultaneity on multisensory gain. Using violations of the race model inequality as an indicator of multisensory gain, they found that despite significant differences in RT to unisensory stimuli that resulted from variations in stimulus intensity (i.e., more intense stimuli resulted in faster RTs), multisensory gain was restricted to a narrow range of SOAs within 50 ms of simultaneity (see also van der Stoep et al. 2015b). Furthermore, the largest multisensory gain occurred at physical simultaneity (SOA = 0 ms). Interestingly, these authors (Leone and McCourt 2013) propose that in daily life, large numbers of physically simultaneous multisensory events are self-generated and occur in near space (see Previc 1998). As a result, throughout development individuals may routinely have access to instances of “multisensory ground truth” (i.e. consistent cross-modal relations between stimulus properties) and thereby establish priors dictating expectations regarding stimulus spatiotemporal characteristics and location (e.g., distance). While Leone and McCourt’s (2013) speculation seems a plausible mechanism, the nervous system may employ to appropriately integrate sensory information even when arriving at sensory periphery at disparate times (i.e., by taking into account self-generated prior), here somewhat surprisingly, we found no correlation between the two derived measures of temporal performance (i.e., TBW and TWFD). Thus, it is possible that sensory expectations or priors of co-occurrence vary differently across external space for perceptual vs. action-based tasks. Future experiments may be designed to test this conjecture.

Taken together, the results seemingly indicate that the SJ and MRT tasks likely index partially dependent multisensory processes that are somewhat dissociable and more readily indexed when considering relatively larger inter-subject variability (see Leone and McCourt 2015; Harrar et al. 2016, for a similar argument). In other words, while an opposing relationship between multisensory binding and gain as a function of distance is not necessarily evidenced in group-level measures, this relation becomes more apparent at an individual subject level (when controlling for intra-subject variability). As argued in Van der Stoep et al. (2016b), multisensory gain is critical for boosting spatial localization when unisensory stimuli are weakly effective, including under circumstances when stimuli are perceived from afar. Conversely, when objects are in close proximity to the body (i.e., peripersonal or reachable space; Serino et al. 2015, 2017), spatial precision may be of less importance. On the other hand, for near stimuli, the timing at which evasive or defensive actions should be started takes priority. Furthermore, as an object moves at a constant speed in the world, the energy it produces traverses the retina at a higher rate when the object is near vs. far. Similarly, for auditory motion, interaural time and level differences (the primary cues for the localization of sounds in azimuth) change more rapidly for near vs. far stimuli. Hence, we speculate that to integrate dynamic auditory and visual stimuli in the world in a similar manner regardless of whether the objects are near or far, multisensory temporal binding may be more liberal (i.e., less acute) in near vs. far space. Stated differently, effective multisensory processing may necessitate a greater temporal window of integration to accumulate sufficient evidence in a region of space with rapidly changing spatial information—the near space. In future work, it will be interesting to test this prediction by probing whether there is a linear relationship between stimulus speed (as measured in visual and auditory angle) and the size of temporal-binding windows.

In sum, the current findings contribute to our understanding of the complex interactions that are continually occurring between the spatial and temporal characteristics of auditory and visual stimuli likely to be associated with the same object or event, with an emphasis on the dimension of depth. At the single subject level, our results illustrate opposing effects of distance on multisensory gain and multisensory temporal binding. However, this pattern of results was not consistent across all temporal intervals, and a direct relationship between temporal binding and multisensory gain at a group level was not apparent. Taken together, these findings seemingly indicate that the neural subsystems responsible for multisensory gain as measured via redundant target tasks and multisensory temporal binding as measured via synchrony judgment tasks are partially dependent but subject to larger inter-individual variability (see Leone and McCourt 2015; Harrar et al. 2016, for a similar argument).

## Electronic supplementary material

Below is the link to the electronic supplementary material.

Supplementary material 1 (DOCX 381 KB)

## Footnotes

1 i.e. MRE above and beyond that predicted by statistical facilitation/independent processing of sensory input (e.g. Miller 1982, 1986, 2015; Ulrich et al. 2007).

2 The SJ task and MRT task were presented in separate blocks, because a pilot study showed that asking participants to both respond as fast as possible to the onset of a stimulus and, after that, report on the simultaneity of stimuli resulted in very slow responses.
