# Supplementary material for: Audiovisual integration in depth: multisensory binding and gain as a function of distance
Source: Exp Brain Res. 2018 Apr 26;236(7):1939–51. doi: 10.1007/s00221-018-5274-7 (PMC6010498; doi:10.1007/s00221-018-5274-7)
Supplement: Supplementary file 1 — Supplementary material 1 (DOCX 381 KB) [file 221_2018_5274_MOESM1_ESM.docx]

**SUPPLEMENTARY MATERIAL:**

**Audiovisual Integration in Depth: Multisensory Binding and Gain as a Function of Distance**

Jean-Paul Noel^1, 2^, Kahan Modi^2^, Mark T. Wallace^2, 3, 4^, Nathan Van der Stoep^5^

^1^ Neuroscience Graduate Program, Vanderbilt Brain Institute, Vanderbilt University Medical School, Vanderbilt University, Nashville, TN 37235, USA

^2^ Vanderbilt Brain Institute, Vanderbilt University Medical School, Vanderbilt University, Nashville, TN 37235, USA

^3^ Department of Hearing and Speech Sciences, Vanderbilt University Medical Center, Nashville, TN 37235, USA

^4^ Department of Psychology, Vanderbilt University, Nashville, TN 37235, USA

^5^ Department of Experimental Psychology, Helmholtz Institute, Utrecht University, The Netherlands

Corresponding Author:

Jean-Paul Noel

[jean-paul.noel@vanderbilt.edu](mailto:jean-paul.noel@vanderbilt.edu)

7110 MRB III BioSci Bldg
PMB 407933
465 21st Ave South
Nashville, TN 37240-7933

**Results**

In the main text we describe race-model violations (RMV) only at the stimulus onset asynchronies (SOA) that demonstrate multisensory enhancement **(rMRE and aMRE; SOA = -150 ms, -50ms, 0 ms, 50 ms, and 150ms**). That is, we adopt a two-step approach in which first we scrutinize response times in a general fashion (i.e., are response times in the multisensory condition faster than the unisensory conditions?), and only in a subsequent step examine the particular quantiles in which violations of the race-model occur. We take this approach as in order for RMV to truly index multisensory integration, RMVs must occur within the context of multisensory facilitation. Thus, by implementing the mentioned two-step approach, we take the most conservative approach possible to describing multisensory integration.

In an addition to the RMV analysis as a function of deciles reported in the main text, RMI violations were equally quantified as the difference in the probability of observing a particular RT between the AV and the race model CDF across the range of RTs (rather than differences in RT across a range of quantiles of the CDFs). Significant violations of the race model inequality occurred **at -150ms, -50ms and 0 ms SOAs. The temporal extent over which these violations occurred was numerically greater for stimuli presented in the far space (SOA_-50_ RT range: 271-369ms, size=98ms; SOA_0_ RT range: 241-337ms, size=96ms) as compared to the near space (SOA_-50_ RT range: 283-367, size=84 ms: SOA_0_ RT range: 240-280ms, size=65ms) for SOAs -50ms and 0ms, but not -150ms (Near: SOA_-150_ RT range: 384-492, size=108 ms**; **Far: SOA_-150_ RT range: 373-465, size=92 ms).**

On the other hand, however, it is equally possible that a particular range of quantiles of RTs show RMV, without the distribution of RTs as a whole demonstrating rMRE/aMRE. Here, thus, for completeness we describe RMVs for all SOAs tested, although we consider “RMVs without aMRE/rMRE” effects to be spurious or a consequence of attentional factors and experimental design. In particular, it must be noted (see below) that RMV at SOA not demonstrating aMRE/rMRE are at late quantiles/slow reaction time ranges. In fact, the effects described below are in response time ranges where the second sensory modality has already appeared, and thus these effects are best understood as priming effects.

Regarding race-model analysis at different quantiles, **in addition to violations at SOA = -150, -50, 0, and 50ms** described in main text, we equally observed violations **at -250ms**. More specifically, in the case of SOA = -250 ms, violations were only present in the near condition, between the 60^th^ and the 80^th^ percentile **(see Figure S1)**.

**Figure S1**. Race model inequality violations as a function of distance (near = grey, far = black) and SO. Significant violations are indicated with an asterisk (*p* < 0.05 corrected for the nine percentiles tests using the Bonferroni method).

When RMI violations were quantified as the difference in probability of observing a particular RT across the range of RTs, rather than differences in RT across a range of quantiles, significant violations of the race model occurred at SOA = -250ms, in addition to those SOAs described in the main text. In the case of SOA = -250 ms, RMV were observed in the range between 475 ms and 574 ms (duration: 99 ms) in the near space and in the range between 506 ms and 523 ms (duration: 17 ms) in the far space (see Figure S2).

**Figure S2.** Race model inequality violations (in percentage; y-axis) as a function of distance (near = grey, far = black), reaction times (x-axis) and SOA. Rectangles under y = 0 indicated RT range in which RMV is significant.
